# BPDGAN: A GAN-Based Unsupervised Back Project Dense Network for Multi-Modal Medical Image Fusion

**DOI:** 10.3390/e24121823

**Published:** 2022-12-14

**Authors:** Shangwang Liu, Lihan Yang

**Affiliations:** 1 College of Computer and Information Engineering, Henan Normal University, Xinxiang 453007, China; 2 Engineering Lab of Intelligence Business & Internet of Things, Xinxiang 453007, China

**Keywords:** unsupervised learning, GAN, multi-modal medical image fusion, dense residual network, convolutional block attention

## Abstract

Single-modality medical images often cannot contain sufficient valid information to meet the information requirements of clinical diagnosis. The diagnostic efficiency is always limited by observing multiple images at the same time. Image fusion is a technique that combines functional modalities such as positron emission computed tomography (PET) and single-photon emission computed tomography (SPECT) with anatomical modalities such as computed tomography (CT) and magnetic resonance imaging (MRI) to supplement the complementary information. Meanwhile, fusing two anatomical images (like CT-MRI) is often required to replace single MRI, and the fused images can improve the efficiency and accuracy of clinical diagnosis. To this end, in order to achieve high-quality, high-resolution and rich-detail fusion without artificial prior, an unsupervised deep learning image fusion framework is proposed in this paper. It is named the back project dense generative adversarial network (BPDGAN) framework. In particular, we construct a novel network based on the back project dense block (BPDB) and convolutional block attention module (CBAM). The BPDB can effectively mitigate the impact of black backgrounds on image content. Conversely, the CBAM improves the performance of BPDGAN on the texture and edge information. To conclude, qualitative and quantitative experiments are tested to demonstrate the superiority of BPDGAN. In terms of quantitative metrics, BPDGAN outperforms the state-of-the-art comparisons by approximately 19.58%, 14.84%, 10.40% and 86.78% on AG, EI, Q_abf_ and Q_cv_ metrics, respectively.

## 1. Introduction

Clinical medical imaging often involves the acquisition of medical images which can characterize different biological information in a variety of modalities. Magnetic resonance imaging (MRI) provides high-resolution information on tissue structure [1], and computed tomography (CT) provides higher resolution in assessing density [2], and single-photon emission computed tomography (SPECT) focuses on functional information on organs and diseased tissues [3], and positron emission computed tomography (PET) reflects the presence or absence of physiological lesions at the molecular level [4,5]. The effective information contained in single-modality images cannot sufficiently meet the information demand for clinical diagnosis. Researchers have attempted to solve this problem through image fusion, and in recent years multimodal medical fusion imaging has attracted a great deal of interest in the clinical field [6,7].

Developing a means to improve the resolution of structural information, while also preserving functional information, is the key problem to be solved in multimodal medical image fusion tasks [8]. With the continuous development of deep learning in recent years, strategies based on convolutional neural networks have gradually highlighted the strengths of image fusion [9]. In fact, most of the existing fusion frameworks are still manually designed rather than employing end-to-end fusion, the former technique being unable to remove the reliance of conventional fusion strategies on a priori knowledge [10]. Therefore, under the generative adversarial network (GAN) framework, we put forward an unsupervised back project dense network for multi-modal medical image fusion. Moreover, the fusion problem in information theory can be almost summarized as the entropy issues in essence. and GAN performs well in this kind of task. Our main contributions are as follows:

1. The BPDB module is proposed and utilized in conjunction with the CBAM module. These modules can eliminate the obstacle of large black backgrounds in the fusion results and obtain high-quality fusion results.
2. An end-to-end multimodal medical image fusion model is put forward to implement the fusion of three kind of medical images with MR images. No manual priori knowledge is required, no labelled data are needed, and the model’s robustness ability is strong.
3. Our loss function, designed for medical image fusion, contains a content loss function and a gradient loss. The gradient loss focuses on high-frequency information of the image. An adversarial mechanism function with gradient information is used to make the fused images texturally clear and content-rich.

## 2. Related Work

With the development of signal processing technologies, more and more image fusion methods have emerged in the past ten years. These methods can be categorized into two types: conventional and deep learning-based methods. Conventional medical image fusion techniques can be divided into two subtypes: spatial domain and frequency domain methods [11]. The spatial domain fusion technique manipulates image pixels directly with simple rules (such as maximum), but is less effective. Because of the spatial domain processing method, it is difficult to decouple high-frequency information and low-frequency (global and detailed) information, leading to increasingly complex fusion rules. In contrast, transform domain fusion methods can fuse images with more high-frequency details. Multi-scale transform (MST) is the standard method for frequency domain-based fusion [12]. The MST-based approach involves three steps: decomposition, fusion rule selection and reconstruction. First, the source image is transformed into the frequency domain, where it is decomposed into a high-frequency sub-band image (HSI) and a low-frequency sub-band image (LSI), with the HIS containing mainly texture details and the LSI containing the image feature distribution and background information. Subsequently, the LSI and HSI are fused using different fusion rules. Finally, the image is reconstructed by the inverse of the decomposition process and transformed into the time domain. Representative examples include the shear wavelet transform [13], Laplace pyramid (LP) transform [14], discrete wavelet transform (DWT) [15], gradient pyramid transform [16], and double tree complex wavelet transform (DTCWT) [17]. However, all these fusion methods contain a down-sampling process, which always harms image information and blunts the texture edges. To address this issue, schemes without a down-sampling process, including the non-sampled contour transform (NSCT) [18] and the non-sampled shear wavelet transform (NSST) [19], have been proposed. For subsequent feature fusion, conventional approaches are limited by their forcing the same transformation to be performed on medical images of different modalities without considering the feature differences among the various modalities, resulting in a poor feature representation. However, the fusion rules are so complex that the algorithm efficiency is low.

In subsequent years, learning-based solutions were proposed to address these issues. Deep learning fusion strategies can be divided into three categories: autocoders (AE), traditional convolutional neural networks (CNN) and generative adversarial networks (GAN). One of the best-known AE-based approaches is DenseFuse [20], which trains the encoder and decoder on the MS-COCO. There is also a classic CNN-based approach, PMGI [21], which implements end-to-end feature extraction, image fusion and reconstruction. FusionGAN [22] pioneered the GAN-based approach to image fusion by building an adversarial mechanism between the fused image and the input image, while training the generator and discriminator to improve the texture detail of the fused image. However, all these existing methods have their own drawbacks. DenseFuse is a universal model, which is trained on a generic dataset and is not suitable for medical image fusion tasks. PCNN [23] relies on a priori knowledge and is less robust. PMGI is an end-to-end model, which does not require a priori knowledge, but the fused images are devoid of details and have serious information loss. Although FusionGAN provides better texture preservation, it uses just one input image to participate in the adversarial mechanism, and the information of the other image is severely lost. Furthermore, these existing methods have poor performances on edge, texture and color (pseudo color with gray) information.

To solve these problems, a back project dense generative adversarial network is proposed in this paper.

## 3. Proposed Method

**Figure 1:**
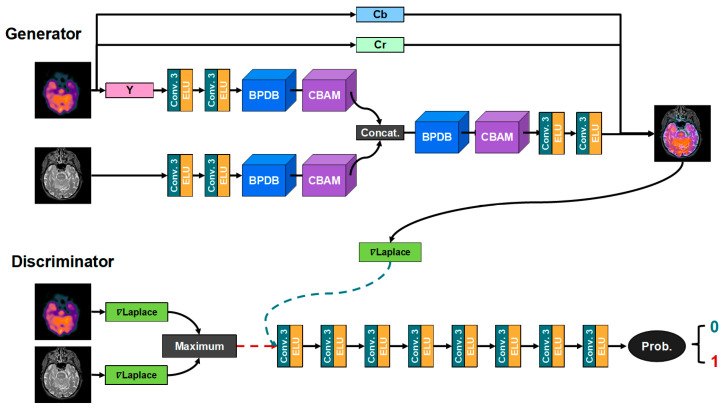
Network structures of back project dense generative adversarial network.

We propose a back project dense generative adversarial network to achieve high-resolution multimodal image fusion, and its pipeline architecture is shown in Figure 1. First, to preserve the functional information in the color images, the PET and SPECT images with RGB color space are first converted into YCbCr color space to separate the luminance channel (Y) from the color channel (Cb, Cr) [24]. Secondly, the Y channel of the image is sent to BPDGAN for fusing with the MR image. Then, the fused Y channel is put out. Finally, the fused Y channel is combined with the Cb and the Cr channels. Then, it is inverse-transformed back into the RGB color space. For single-channel CT images, the image can be fed directly into BPDGAN for fusion with the MR image.

In medical image fusion tasks, the image to be fused often has a large black background, which can cause a loss of overall brightness and blurred edges, reducing visual quality and texture detail. In order to reduce the negative impact of the black background on the fusion result, the back project dense block (BPDB) module is introduced in this paper, where the feature map is fed into the back proje module and passes through three modules: the feature enhancement module, the residual calculation module and the feature reconstruction module. The input features undergo a series of calculations to obtain the residuals and add the enhanced feature map. BPDB and the convolutional block attention module (CBAM) work together to reduce the negative impact of the black background.

In this paper, we also design a generator loss function that matches the medical image fusion task to jointly prompt the generator so as to ensure the generator produces high-quality fused images. Meanwhile, our model establishes an adversarial mechanism between the generator and the discriminator to jointly constrain the generator and the discriminator. Via the adversarial mechanism, the fused image gradient gets closer and closer to the joint gradient of the input image until the discriminator is confused. The texture detail can be further enhanced.

### 3.1. Pre-Processing

The PET images involved in the fusion are pseudo-color images with functional information presented in RGB color space, while the MRI images are single-channel images, which have only structural information. In order to protect the functional information of the PET and SPECT images, we choose to pre-process the PET and SPECT images to YCbCr color space [25], separating the color information (Cb and Cr channels) from the luminance information (Y channel) and using only the luminance information (Y channel) for fusion.

During the fusion procedure, only the MRI image and the Y component of the PET image are processed, both of which are grayscale images, and the color information in the PET image will be perfectly preserved.

### 3.2. Network Structures

To enhance the texture detail of the fused images and to preserve the structural information that the Y-channel of the PET image has been integrated with the MRI image, the BPDGAN adversarial generation network architecture is proposed and described as follows.

#### 3.2.1. Generative Adversarial Networks

Labeling medical image requires specialized expertise, and it is too expensive and time-consuming to label them by hand. Therefore, a generative adversarial network (GAN) [26] is employed in this paper.

The GAN architecture contains a generator (G) and a discriminator (D). During training period, the parameters of G are updated not from data samples, but from the back propagation of D. Thus, the GAN-based network architecture can be freed from the labeling data.

G is trained to generate realistic samples from random noise or latent variables that can be formulated as *x* = *G*(*Z*). For the fusion task, the generator has two inputs and its training goal is to make the generated data as close as possible to the real data *P_data_*(*x*, *y*). The optimization process for the generator can be executed by Equation (1).

(1) $$G^{*}=\arg\underset{G}{\min}\;Div(P_{G}(z_{1},z_{2}),P_{data}(x,y)),$$

where $Div(P_{G}(z_{1},z_{2}),P_{data}(x,y))$ represents the distance between the generated data distribution $P_{G}(z_{1},z_{2})$ and the true data $P_{data}(x,y)$.

Since both the generating distribution and the true distribution are unknown, *D* is trained simultaneously to identify the truthfulness of the generated data, as shown in Equation (2).

(2) $$V(G,D)=E_{x,y∼p_{data}(x,y)}[\log D(x,y)]+E_{x,y∼p_{G}(z_{1},z_{2})}[\log(1-D(x,y))],$$

The higher the discriminator scores are for data sampled from the true data $P_{data}(x,y)$, the lower the generator scores are for data sampled from the generated distribution $P_{G}(z_{1},z_{2})$, both of which maximize the function V(G,D). For more detail, please refer to Equation (3).

(3) $$D^{*}=\arg\underset{D}{\max}\;V(G,D),$$

During the training process of the network, *G* and *D* keep playing a max and min mechanism, respectively. At first, the direct interval distance between the generated data of the generator and the real data distribution is large. However, as the number of iterations increases, the two distributions become closer and closer until the discriminator cannot distinguish the generated data from the real data, and a Nash equilibrium is reached between the generator and the discriminator. At this point, the generator training is completed, and the generated data can be regarded as the real data.

#### 3.2.2. Overall Network Architecture

The BPDGAN network structure is elaborately designed to fuse the PET’s Y-channel with the grayscale image to obtain a richer detailed texture with higher image resolution. Our model improves the medical image fusion in terms of both the network structure and the loss function. Large black areas of multimodal medical images (e.g., PET) can reduce the brightness of the fused image and blur the texture structure of the image boundaries. To address the problem, we improve the network structure and propose a back-projection module (see Figure 1) for removing the obstacle of black background on the feature subtraction fusion results. For the loss function, the model dynamically adjusts the loss function to optimize the prediction results and uses gradient loss to enhance the fused image detail performance.

To meet the high accuracy needs of medical image diagnosis for lesion texture, we introduce an attention mechanism into our model. The attention module assigns different weights to each part of the input information, enabling the model to extract the more important features. The model focuses its limited computational resources on the important features to avoid information overload and optimize the fusion results.

To further enhance the fused image detail texture, we build an adversarial mechanism based on gradients. The real data are the joint gradient map of the two input images, which is computed by the Laplace operator as well as the maximax principle; and the pseudo data are the gradient map of the fused image, which is computed by the Laplace operator. Within the discriminator, the real data and the pseudo data undergo continuous adversarial learning. Thus, the generator is forced to produce fused images with richer texture detail and more realistic gradients. The GAN can be optimized in the way as shown in Equation (4).

(4) $$\underset{G}{\min}\;\underset{D}{\max}\;Var_{GAN}(G,D)=E_{x,y∼p_{data}(x,y)}[\log D(\nabla x,\nabla y)]+E_{a,b∼p_{z}(a,b)}[\log(1-D(\nabla G(a,b)))],$$

#### 3.2.3. Generator Architecture

The structure of the generator of our model is shown in Figure 1; the PET’s Y-channel and the MRI image are fed into the network through two branches with the same structure. Taking the MRI image as an example, the MRI image first enters the two convolution–activation modules for feature extraction, where the convolution layer uses a 3 × 3 convolution kernel and the activation method uses ELU. The extracted features are formulated as follows:

(5) $$F_{1}=H_{ext}\left(I\right),$$

**Figure 2:**
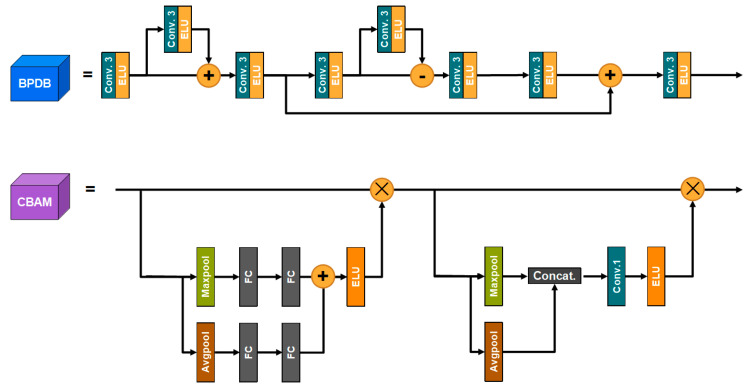
Structures of BPDB and CBAM.

The extracted high-dimensional features then enter the back-projection module (BPDB), details of which are described in Section 3.2.5. The features after going through the BPDB can be represented as:

(6) $$F_{2}=H_{BPDB}\left(F_{1}\right),$$

where $H_{BPDB}$ represents the back-projection function. The model feeds the back-projected features into the attention module for weighting, which is shown in Figure 2 and discussed in detail in Section 3.2.6. The weighted feature $F_{2}$ can be formulated as follows:

(7) $$F_{3}=H_{CBAM}\left(F_{2}\right),$$

$H_{CBAM}$ is the operator function of the attention mechanism, which contains channel attention and spatial attention operations. Afterward, the two branches are merged into one by concat the operation, and the fusion feature can be expressed as follows:

(8) $$F_{fusion}=H_{concat}(F_{MRI,3},F_{\mathrm{PET}\_Y,3}),$$

where $H_{concat}$ indicates the feature map stitching operation. Afterwards feature $F_{fusion}$ is again back-projected and weighted, and the output features can be expressed as follows:

(9) $$F_{Enhance}=H_{\;Composite}\left(F_{fusion}\right),$$

where $H_{\;Composite}$ denotes the composite function of the feature back-projection and the attention module.

The final fused image is obtained after two convolution–activation modules, and the convolution layer uses 3 × 3 convolution, and the activation method is ELU.

#### 3.2.4. Discriminator Architecture

The discriminator of our model BPDGAN is also shown in Figure 1, whose input is not the image itself, but the image gradient. The two images to be fused are calculated using the Laplace operator to yield two gradient maps, which are then calculated according to the principle of maximum to obtain the joint gradient map, i.e., the real data. The fused image is calculated by using the Laplace operator to generate the fused gradient map as the false data. The features extracted after the convolution enter the probability module, which is utilized to calculate the probability value to determine whether the generated data are real or not.

#### 3.2.5. Back Project Dense Block (BPDB)

The inverse projection module is also elaborately designed to leverage feature operations to reduce the negative effect of large black backgrounds on the fusion results. The structure is shown in Figure 2. The feature map is fed into the reverse projection module and passes through three modules, where convolution–activation layer has a convolution kernel size of 3 × 3 and the activation method is ELU. First is the feature enhancement module, in which feature *F_pre_* enters the module and is convolution-activated to encode the feature *F_enc_*; and then *F_enc_* enters the difference calculation module, whose output value is added to *F_enc_* and sent to the convolution–activation module to decode *F_add_*. The residual calculation module is similar to the feature enhancement module. However, instead of adding the encoded features and the difference calculated features, the absolute value is then subtracted and fed into the decoder to obtain the residual features *F_res_*. In the final feature reconstruction module, *F_residual_* is encoded to obtain *F_res_en_*, which is added to and decoded to output feature *F*.

#### 3.2.6. Convolutional Block Attention Module (CBAM)

In contrast to the traditional approach to computing 3D attention maps, CBAM divides attention into channel attention and spatial attention. The architecture is shown in Figure 2. Feature F is first fed into the channel attention module, where it is copied to two branches and subjected to maximum pooling and average pooling to yield F_max_ and F_avg_. F_max_ and F_avg_ are fed into the fully connected layer to obtain the channel weights, which are then summed and activated by the module (the activation method is ELU) to generate the channel attention. The channel attention weighted feature F_1_ is fed into the spatial attention module and copied to both branches for maximum pooling and average pooling to obtain F_1_max_ and F_1_avg_. F_1_max_ and F_1_avg_ are spliced to yield F_concat_, and the spliced features are then fed into the 1 × 1 convolutional layer and ELU activation layer to generate spatial attention.

### 3.3. Loss Function

For the special requirements of texture details for multimodal medical image fusion tasks, we design a series of semi-supervised loss functions, which include generator loss and discriminator loss.

#### 3.3.1. Generator Loss

The loss function of the generator is based on adversarial loss, pixel-level Euclidean loss and texture loss, which can be calculated from Equation (10).

(10) $$L=L_{Gan}+\lambda_{1}L_{pixel}+\lambda_{2}L_{grad},$$

where $L_{Gan}$ ismeans the adversarial loss of the generative adversarial network; $L_{pixel}$ is the absolute pixel distance loss optimized using the screening map; and $L_{grad}$ denotes the gradient loss based on the gradient map. $\lambda_{1}$ and $\lambda_{2}$ are the weights of pixel distance loss and texture loss, respectively, which ensure that the three loss functions are of equal importance.

#### 3.3.2. Adversarial Loss

In order for the generator to generate images closer to the ideal fused image, the loss needs to be built between the generator and the discriminator. The traditional adversarial loss reduces the max–min problem to log(1−D(G(I))). However, at the beginning of the training phase, log(1−D(I)) may saturate, and the generator network is trained using D(G(I)) maximization. To provide a stronger gradient, a square operation is added to the maximization operation. The definition of $L_{Gan}$ is as follows.

(11) $$L_{Gan}=\frac{1}{N}\sum_{n=1}^{N}{\left(D(\nabla G(M_{n},I_{n}))-c\right)}^{2},$$

where *N* is the number of images in one batch during the training period; *c* is the alteration rate label for the discriminator to identify true and false images, and here *c* = 1; ∇ denotes the Laplace operator for the gradient map calculation; M,I denote the input MRI image and CT or Y-channel for PET and SPECT.

#### 3.3.3. Pixel-Level Euclidean Loss

Once the pixels at the corresponding positions of the input image and the fused image are taken, their absolute distances can be calculated; the smaller the distance is, the closer the intensity of the two images is (see Equation (12)).

(12) $$L_{pixel}=\frac{1}{X\times Y}\sum_{x=1}^{X}\sum_{y=1}^{Y}Map_{1}{\left(G(M_{x,y},I_{x,y})-M_{x,y}\right)}^{2}+Map_{2}{\left(G(M_{x,y},I_{x,y})-I_{x,y}\right)}^{2},$$

where *x*, *y* denote the pixel values of the *x*th row and *y*th column; *X*, *Y* are the height and width of the image, respectively; $Map_{1}$ and $Map_{2}$ represent the filtered maps generated by the judgment block based on the two input images.

#### 3.3.4. Gradient Loss

The gradient of the image can partially characterize the texture details, more so for the contrast sharp MRI images, thus requiring the fused image to have a similar gradient to the input image. Combined with the screening map, the gradient loss is computed as follows.

(13) $$L_{grad}=\frac{1}{H\times W}\sum_{h=1}^{H}\sum_{w=1}^{W}Map_{1}{\left(\nabla G(M_{h,w},Y_{h,w})-\nabla M_{h,w}\right)}^{2}+Map_{2}{\left(\nabla G(M_{h,w},Y_{h,w})-\nabla Y_{h,w}\right)}^{2},$$

#### 3.3.5. Discriminator Loss

Not only the generator needs the loss function to optimize the quality of the fused image, but also the discriminator needs the loss function to accurately identify the true and false fused images. In this paper, a loss function based on the gradient map is designed for the discriminator, where the “false data” is the gradient map of the fused image, which can be calculated from Equation (14).

(14) $$Grad_{1}=abs(\nabla G(M,I)),$$

The “true data” required by the discriminator comes from the joint gradient map constructed by *M* and *I*, which can be calculated from Equation (15).

(15) $$Grad_{2}=\max imum(abs(\nabla M),abs(\nabla I)),$$

where *abs* denotes the absolute value function; and *maximum* represents the maximization function.

Based on the two gradient plots mentioned above, the loss can be computed from Equation (16).

(16) $$L_{D}=\frac{1}{X}\sum_{x=1}^{X}{\left(D(Grad_{1}{}^{x})-a\right)}^{2}+{\left(D(Grad_{2}{}^{x})-b\right)}^{2},$$

where *a* is the label of “false data” which is set to 0; and b is the label of “true data” which is set to 1. This causes the discriminator to treat the joint gradient map of the image as true data and the gradient map of the fused image as false data. This constraint guides the generator to adjust $Grad_{1}$ based on $Grad_{2}$ to enhance the texture of the fused image in the confrontation.

## 4. Experimental Results and Analysis

To verify the superiority of our BPDGAN in multi-modal medical image fusion, a number of experiments were conducted on publicly available datasets.

### 4.1. Training Details

The PET and MRI images employed in this experiment were obtained from the publicly available datasets at the Harvard Medical School website. It should be noted that our model does not require labeling data for training, and these datasets are only used to verify the validity of our model. PET images are three-channel pseudo-color images of 256 × 256 size, and MRI images are single-channel grayscale images of the same size.

During adversarial training, the batch-size is set to b and one iteration is divided into K steps. The total number of trainings is M times, and the discriminator is trained p times more often than the generator. After extensive experiments on the model, the hyperparameters were set as follows: b = 32, p = 2, M = 300, and the other parameters were updated using the Adam Optimizer.

The hardware environment sets as follows: CPU AMD R5 5600X, GPU RTX-3060 (12G). The software environment sets as follows: OS, Windows 10; programe language, Python 3.7.6; deep learning framework, Pytorch 1.10.0. The ratio of training set, validation set and test set of the dataset is 7:2:1, and the specific training process is shown in Algorithm 1.
**Algorithm 1:** Generative adversarial network training algorithm (Take SPECT as an example)Require: MR($I_{M}$) and PET($I_{P}$) image;Require: A generator G and a discriminator D;Require: Initialize parameters θg and θd randomly;for number of training iterations **M**   for k step:   RGB2YCbCr($I_{P}$)→Y, Cb, Cr   G($I_{M}$,Y)→$Y_{F}$      YCbCr2RGB($Y_{F}$, Cb, Cr)→F   End Calculate $L=L_{Gan}+\lambda_{{}_{1}}L_{pixel}+\lambda_{2}L_{texture}$
Update θd by $L_{Gan}$: θd←AdamUpdate θg by L: θg←Adam**Return:** Trained generator

### 4.2. Quantitative Evaluation Indicators

Four evaluation indicators are adopted in this paper. These are *Q_abf_*, *Q_cv_*, *AG* and *EI*. The *Q_abf_* focuses on local information and uses local information to measure the ability of the fused image to preserve important information of the input image [27]. The *Q_abf_* can be used to gauge the quality of the fused image, as shown in Equation (17).

(17) $$Q(A,B,F)=\frac{1}{\left|W\right|}\sum_{\omega \in W}\left(\lambda (\omega )Q_{0}(A,F|\omega )+(1-\lambda (\omega ))Q_{0}(B,F|\omega )\right),$$

where *W* is used to divide the local area; λ(ω) represents the weights of local area; A,B are the input images; *F* is the fused image.

The quality of the local area image can be expressed as *Q_cv_*. It calculates the mean square error of the weighted difference image between the fused area image and the source area image, and the quality of the fused image is a weighted sum of the local area image quality measures [28]. The *Q* equation is formulated as follows.

(18) $$Q=\frac{\sum_{\omega \in W}\left(\lambda (A_{\omega })D(\omega_{1},F|\omega )+\lambda (B_{\omega })D(B_{\omega },F|\omega )\right)}{\sum_{\omega \in W}\left(\lambda (A_{\omega })+\lambda (B_{\omega })\right)},$$

where *D* is the local similarity function.

*AG* (average gradient) can reflect the image’s ability to represent details and textures and is often used to quantify the sharpness of an image [29]. For an image with size *H* × *W*, its average gradient can be computed from Equation (19).

(19) $$AG=\frac{1}{\left(H-1)(W-1\right)}\sum_{h=1}^{H}\sum_{w=1}^{W}\frac{1}{4}\sqrt{{\left(\frac{\partial g(h,w)}{\partial h}\right)}^{2}+{\left(\frac{\partial g(h,w)}{\partial w}\right)}^{2}},$$

where (h,w) represents the image coordinates; and $\frac{\partial g}{\partial h},\frac{\partial g}{\partial w}$ indicates the gradient information of image vertical and horizontal. The average gradient value reflects the amount of information contained in the image, and it can evaluate the fusion effect.

*EI* (edge intensity) represents the image quality and sharpness; the edge strength shows a positive correlation with the sharpness of the image [30]. For an image *F* with size *H × W*, whose edge intensity can be calculated from Equation (20).

(20) $$EI=\frac{\sqrt{\sum_{h=1}^{H}\sum_{w=1}^{W}s_{x}{\left(h,w\right)}^{2}+s_{y}{\left(h,w\right)}^{2}}}{H*W},$$

where

(21) $$s_{x}=F*h_{x},s_{y}=F*h_{y},$$

where $h_{x}$ and $h_{y}$ are the Sobel operators in the *x* and *y* directions, respectively.

### 4.3. Quantitative and Qualitative Comparison Results

To verify the effectiveness of our BPDGAN for multi-modal image fusion, five state-of-the-art methods, DDcGAN [31], DenseFuse, GCF [32], IFCNN [33], PMGI and U2Fusion [34] are employed to compare with our approach in this paper.

In order to solve the brightness destruction and edge blurring problems brought by large black backgrounds, a reverse projection module is introduced to mitigate the negative impact of invalid information on fusion by using residual operations. Qualitative results show that BPDGAN has clear details, high image quality, significant edge contrast, and no loss of luminance information.

**Figure 3:**
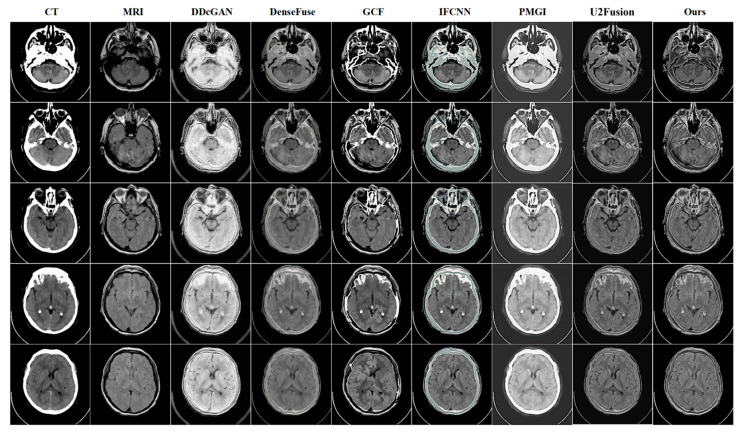
Fusion result of CT-MRI.

In the CT-MRI fusion task shown in Figure 3, the DDcGAN texture is missing, the gray value is too large, and the information is incompletely preserved. DenseFuse has blurred edges and little texture detail. MRI information is lost in the white area of GCF. In IFCNN, some fields of the original CT image are poorly fused. A large amount of MRI information is lost in PMGI, and the visual effect is poor. In CT-MRI tasks, DDcGAN has poor visual quality and serious loss of MRI information. Distinct from other approaches, a structural loss function and a gradient-based adversarial loss function are put forward to protect high-frequency information and texture gradient information, respectively, and enhance the fused images by nonlinear loss constraints; and the BPDGAN high-frequency information performs the best in the three tasks. Beside these qualitative experiments, quantitative evaluation is carried out too, and the fused images are judged quantitatively from the data, and the results of CT-MRI are shown in Table 1.

**Figure 4:**
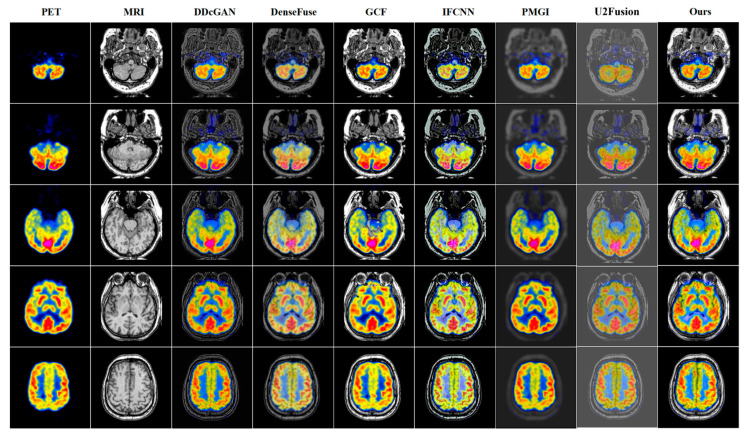
Fusion result of PET-MRI.

The results of the PET-MRI fusion task are shown in Figure 4. From Figure 4, it can be clearly seen that the grayscale information of DDcGAN, DenseFuse, IFCNN, PMGI and U2Fusion are all corrupted to different degrees, with poor visual effects and imperfect preservation of texture features. Although GCF protects the brightness of the fusion result, the fused image has blurred fused edges and poor quality compared with our BPDGAN. Taking PET-MRI task as example, BPDGAN has the least luminance loss and the most perfect texture. The network also incorporates a CBAM module to increase the focus of lesion information and speed up the training, which can localize lesions more accurately. Previous methods are used to sharpen edges by direct target enhancement and gradient. A quantitative comparison of PET-MRI results is shown in Table 2.

**Figure 5:**
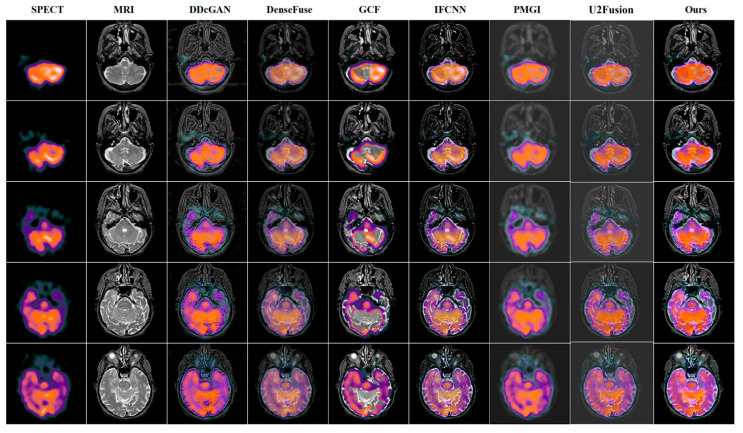
Fusion result of SPECT-MRI.

The results of SPECT-MRI fusion tasks are shown in Figure 5. In Figure 5, the DDcGAN, during the fusion process, will destroy the spectrum, and the edge gradient is not obvious. The fused images of DenseFuse have low brightness and poor visual effect. GCF has better color preservation, but there are noise blocks in the image and structural information is lost, which is unacceptable in medical images. IFCNN is blurred near the boundary line, losing details, and the focus is not prominent, factors which will easily lead to clinical misjudgment. PMGI fusion suffers from serious background defocusing and loses a great deal of structural information while its functional information is well preserved, but the overall is too blurred to be used practically. A quantitative comparison of SPECT-MRI results is shown in Table 3.

It can be seen that, for the PET-MRI fusion task and the SPECT-MRI fusion task, our BPDGAN outperformed the remaining five comparison methods in all five metrics, and BPDGAN had the best combined metrics for the CT-MRI task. The EI index of BPDGAN outperformed the rest of the algorithms in all three modes, and the EI index in the PET-MRI task, for example, was 10.79% higher than the state-of-the-art comparison, which indicated that the model had a clear edge texture, which fully validated the role of BPDB in eliminating the negative effects of a black background. It was the specific structural and adversarial loss functions that gave BPDGAN a clearer gradient texture, and the average gradient was reflected by the AG index, which was 9.6% higher than the state-of-the-art comparison in the CT-MRI fusion task. The Q_cv_ index is based on the regional mean square error of the human visual system (HVS); with the benefit of the CBAM module, BPDGAN can adaptively determine the pixel weights to improve the regional similarity and thus the visual quality of the fusion results. The Q_cv_ metric of BPDGAN was lower than the second one by 68.2%, which proves its higher regional similarity and stronger human eye perception capabilities than other methods. Our model adopted the ground pixel-scale control strategy, and the Euclidean distance between pixels was controlled well, so the pixel-level fusion index Q_abf_ of our model was the highest in PET and SPECT tasks and second only to GCF in CT task. In short, the visual information of our model was perfect, and the pixel-level gap between fused image and source image was small.

### 4.4. Ablation Experiments

We study the effect of different combinations of modules and different combinations of loss functions on our network. For different combinations of modules, the results are shown in Table 4.

For different combinations of loss functions, the results are shown in Table 5. In both sets of ablation experiments, all evaluation metrics showed substantial improvement.

From the above two sets of experiments, it can be seen that our work has yielded very effective results. In the architecture ablation experiment, the evaluation metrics (taking AG as an example) were 6.38% and 2.55% higher with the addition of BPDB and CBAM, respectively, compared to the values registered with the addition of BackBone only. The AG was 9.06% higher with the addition of BPDB and CBAM. This proved that our designed solution performs well in terms of edge, texture and fusion quality. In the loss function ablation experiment, the AG was 3.31% and 2.50% higher with the addition of pixel loss and gradient loss compared to the addition of pixel loss and gradient loss alone. These results demonstrate that *L_pixel_* improved pixel-level performance and *L_grad_* improved more fusion performances with our tactic.

### 4.5. Future Direction: BPDGAN vs. SwinFusion

**Figure 6:**
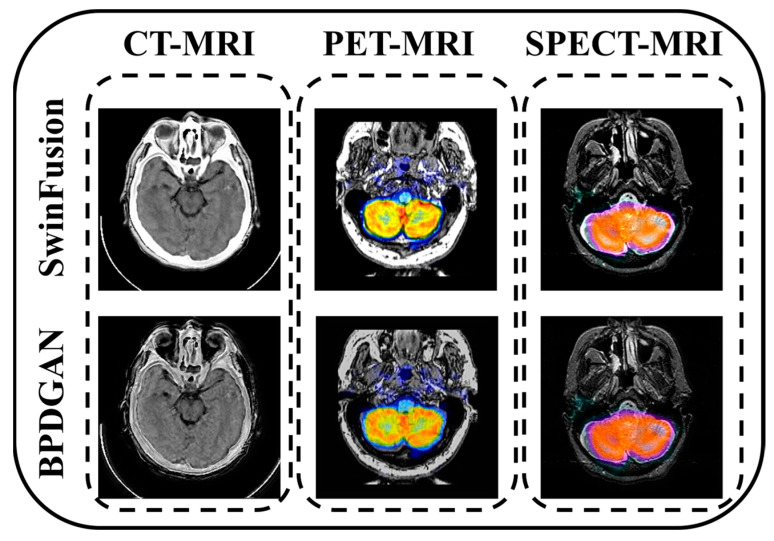
Fusion results of SwinFusion and BPDGAN.

Recently, Jiayi Ma [35] proposed a novel transformer-based long-range learning multimodal fusion pipeline named SwinFusion. In order to compare BPDGAN with the SwimFusion, we also conducted the following comparisons, as shown in Figure 6 and Table 6. These results demonstrate that SwinFusion performs better than our BPDGAN, while SwinFusion has large parameters and trains on large dataset. In the model aspect, we find that transformer-based methods can better focus on the overall information. This is our further improvement direction. For the data aspect, the existing work [36] shows that the quality of the dataset plays an important role in the whole issue. The SwinFusion vs. BPFGAN comparisons also prove this point.

## 5. Conclusions

In this paper, we present the BPDGAN for multimodal medical image fusion. Unlike previous direct fusion approaches, firstly, we leveraged a YCbCr-based color space approach to achieve fusion of texture structure information without the loss of spectral information. Secondly, in the feature fusion stage, BPDB was proposed to reduce the negative effects, such as overall brightness reduction and blurred edges. These qualities were brought by black background to the fused image. Meanwhile, CBAMs were combined to make our model focus more on the location of the lesion and its structure rather than on the large black background. Finally, the model was trained in an end-to-end manner and did not rely on artificial a priori knowledge at all. We conducted both quantitative and qualitative experiments to demonstrate the superiority of BPDGAN against state-of-the-art methods. In future, we will focus on the study of novel attentional mechanisms of clinical importance and then continue to further improve the performance of BPDGAN.

## Figures and Tables

**Table 1:** Quantitative comparison of CT-MRI results.

CT-MRI	DDcGAN	DenseFuse	GCF	IFCNN	PMGI	U2Fusion	Ours
AG↑	9.0754	5.6772	9.1627	7.7264	7.895	6.8957	10.1333
EI↑	91.0206	57.5941	97.2749	81.7838	79.8071	70.5399	99.7997
Q_abf_↑	0.3563	0.3393	0.5682	0.5122	0.508	0.4102	0.5402
Q_cv_↓	4974.886	2599.490	4902.781	1922.205	1592.081	2610.5305	3376.064

**Table 2:** Quantitative comparison of PET-MRI results.

PET-MRI	DDcGAN	DenseFuse	GCF	IFCNN	PMGI	U2Fusion	Ours
AG↑	6.0128	4.9272	7.0089	6.9044	2.7804	3.8598	8.3813
EI↑	60.3775	51.1228	75.6065	73.8009	29.6103	40.6413	84.7561
Q_abf_↑	0.3655	0.2863	0.5848	0.5026	0.1736	0.1705	0.6456
Q_cv_↓	1577.441	947.514	718.393	412.393	3667.634	1861.1615	220.779

**Table 3:** Quantitative comparison of SPECT-MRI results.

SPECT-MRI	DDcGAN	DenseFuse	GCF	IFCNN	PMGI	U2Fusion	Ours
AG↑	4.7542	3.4322	5.9096	6.1458	1.9178	3.8571	6.1660
EI↑	50.1068	36.6158	62.9763	64.8819	20.7479	39.1411	65.3377
Q_abf_↑	0.2334	0.1444	0.3896	0.4065	0.0798	0.2113	0.4353
Q_cv_↓	844.875	560.090	317.356	1009.200	2473.292	886.6785	178.168

**Table 4:** Architecture ablation experiments.

Architecture	AG↑	EI↑	Q_abf_↑	Q_cv_↓
Backbone	7.6270	77.5518	0.3635	2009.348
Backbone + BPDB	8.1466	81.4506	0.5358	417.2723
Backbone + CBAM	7.8281	78.7384	0.4325	620.3889
Backbone + BPDB + CBAM	8.3813	84.7561	0.6456	220.779

**Table 5:** Loss function ablation experiment.

Architecture	AG↑	EI↑	Q_abf_↑	Q_cv_↓
L_Gan_ + L_gard_	8.10	79.8556	0.5264	449.0945
L_Gan_ + L_pixel_	8.17	81.2722	0.4428	727.8431
L_Gan_ + L_pixel_ + L_grad_	8.38	84.7561	0.6456	220.779

**Table 6:** Quantitative comparison of SwinFusion vs. BPDGAN results.

Method		AG↑	EI↑	Q_abf_↑	Q_cv_↓
CT-MRI	SwinFusion	9.2796	94.1302	0.6059	2100.6508
	BPDGAN	10.1333	99.7997	0.5402	3376.064
PET-MRI	SwinFusion	10.7131	110.8256	0.7020	305.3309
	BPDGAN	8.3813	84.7561	0.6456	220.779
SPECT-MRI	SwinFusion	9.9039	98.4848	0.7175	257.2418
	BPDGAN	6.1660	65.3377	0.4353	178.168

## Data Availability

The dataset is available at http://www.med.harvard.edu/AANLIB/home.html, and accessed on 16 July 2021.

## References

[1] Terreno E., Castelli D.D., Viale A., Aime S. (2010). Challenges for Molecular Magnetic Resonance Imaging. Chem. Rev..

[2] Buzug T.M. (2011). Computed Tomography. Springer Handbook of Medical Technology.

[3] Holly T.A., Abbott B.G., Al-Mallah M., Calnon D.A., Cohen M.C., DiFilippo F.P., Ficaro E.P., Freeman M.R., Hendel R.C., Jain D. (2010). Single photon-emission computed tomography. J. Nucl. Cardiol..

[4] Vita T., Okada D.R., Veillet-Chowdhury M., Bravo P.E., Mullins E., Hulten E., Agrawal M., Madan R., Taqueti V.R., Steigner M. (2018). Complementary Value of Cardiac Magnetic Resonance Imaging and Positron Emission Tomography/Computed Tomography in the Assessment of Cardiac Sarcoidosis. Circ. Cardiovasc. Imaging.

[5] Huo X., Deng Y., Shao K. (2022). Infrared and Visible Image Fusion with Significant Target Enhancement. Entropy.

[6] Ma X., Wang Z., Hu S., Kan S. (2022). Multi-Focus Image Fusion Based on Multi-Scale Generative Adversarial Network. Entropy.

[7] Hermessi H., Mourali O., Zagrouba E. (2021). Multimodal medical image fusion review: Theoretical background and recent advances. Signal Process..

[8] Liu Y., Zhou D., Nie R., Hou R., Ding Z. (2020). Construction of high dynamic range image based on gradient information transformation. IET Image Process..

[9] Yousif A.S., Omar Z., Sheikh U.U. (2021). An improved approach for medical image fusion using sparse representation and Siamese convolutional neural network. Biomed. Signal Process. Control..

[10] Hou R., Zhou D., Nie R., Liu D., Ruan X. (2019). Brain CT and MRI medical image fusion using convolutional neural networks and a dual-channel spiking cortical model. Med. Biol. Eng. Comput..

[11] Yang D., Hu S., Liu S., Ma X., Sun Y. (2018). Multi-focus image fusion based on block matching in 3D transform domain. J. Syst. Eng. Electron..

[12] Li G., Lin Y., Qu X. (2021). An infrared and visible image fusion method based on multi-scale transformation and norm optimization. Inf. Fusion.

[13] Deng Y., Wu Z., Chai L., Wang C.-Y., Yamane K., Morita R., Yamashita M., Zhang Z. (2005). Wavelet-transform analysis of spectral shearing interferometry for phase reconstruction of femtosecond optical pulses. Opt. Express.

[14] Wang Z., Cui Z., Zhu Y. (2020). Multi-modal medical image fusion by Laplacian pyramid and adaptive sparse representation. Comput. Biol. Med..

[15] Shensa M. (1992). The discrete wavelet transform: Wedding the a trous and Mallat algorithms. IEEE Trans. Signal Process..

[16] Petrovic V., Xydeas C. (2004). Gradient-Based Multiresolution Image Fusion. IEEE Trans. Image Process..

[17] Selesnick I., Baraniuk R., Kingsbury N. (2005). The dual-tree complex wavelet transform. IEEE Signal Process. Mag..

[18] Lian X.Q., Ding X.H., Guo D.H. (2007). Digital watermarking based on non-sampled contourlet transform. International Workshop on Anti-Counterfeiting, Security and Identification(ASID).

[19] Reddy S., Krishnaiah R.V., Rao Y.R. An Effective Approach in Fusion of Multispectral Medical Images Using Convolution Structure Sparse Coding. Proceedings of the 2021 6th International Conference on Communication and Electronics Systems (ICCES).

[20] Li H., Wu X.-J. (2018). DenseFuse: A Fusion Approach to Infrared and Visible Images. IEEE Trans. Image Process..

[21] Zhang H., Xu H., Xiao Y., Guo X., Ma J. (2020). Rethinking the Image Fusion: A Fast Unified Image Fusion Network based on Proportional Maintenance of Gradient and Intensity. Proc. AAAI Conf. Artif. Intell..

[22] Ma J., Yu W., Liang P., Li C., Jiang J. (2018). FusionGAN: A generative adversarial network for infrared and visible image fusion. Inf. Fusion.

[23] Wang X., Li Z., Kang H., Huang Y., Gai D. (2021). Medical Image Segmentation using PCNN based on Multi-feature Grey Wolf Optimizer Bionic Algorithm. J. Bionic Eng..

[24] Han D., Li L., Guo X., Ma J. (2021). Multi-exposure image fusion via deep perceptual enhancement. Inf. Fusion.

[25] Alwan Z.A., Farhan H.M., Mahdi S.Q. (2020). Color image steganography in YCbCr space. Int. J. Electr. Comput. Eng. (IJECE).

[26] Gui J., Sun Z., Wen Y., Tao D., Ye J. (2021). A Review on Generative Adversarial Networks: Algorithms, Theory, and Applications. IEEE Trans. Knowl. Data Eng..

[27] Xydeas C., Petrović V. (2000). Objective image fusion performance measure. Electron. Lett..

[28] Chen H., Varshney P.K. (2007). A human perception inspired quality metric for image fusion based on regional information. Inf. Fusion.

[29] Cui G., Feng H., Xu Z., Li Q., Chen Y. (2015). Detail preserved fusion of visible and infrared images using regional saliency extraction and multi-scale image decomposition. Opt. Commun..

[30] Rajalingam B., Priya R. (2018). Hybrid multimodality medical image fusion technique for feature enhancement in medical diagnosis. Int. J. Eng. Sci..

[31] Ma J., Xu H., Jiang J., Mei X., Zhang X.-P. (2020). DDcGAN: A Dual-Discriminator Conditional Generative Adversarial Network for Multi-Resolution Image Fusion. IEEE Trans. Image Process..

[32] Xu H., Fan F., Zhang H., Le Z., Huang J. (2020). A Deep Model for Multi-Focus Image Fusion Based on Gradients and Connected Regions. IEEE Access.

[33] Zhang Y., Liu Y., Sun P., Yan H., Zhao X., Zhang L. (2019). IFCNN: A general image fusion framework based on convolutional neural network. Inf. Fusion.

[34] Xu H., Ma J., Jiang J., Guo X., Ling H. (2020). U2Fusion: A Unified Unsupervised Image Fusion Network. IEEE Trans. Pattern Anal. Mach. Intell..

[35] Ma J., Tang L., Fan F., Huang J., Mei X., Ma Y. (2022). SwinFusion: Cross-domain Long-range Learning for General Image Fusion via Swin Transformer. IEEE/CAA J. Autom. Sin..

[36] Roccetti M., Delnevo G., Casini L., Cappiello G. (2019). Is bigger always better? A controversial journey to the center of machine learning design, with uses and misuses of big data for predicting water meter failures. J. Big Data.

